# Roles of Bacterial Mechanosensitive Channels in Infection and Antibiotic Susceptibility

**DOI:** 10.3390/ph15070770

**Published:** 2022-06-21

**Authors:** Margareth Sidarta, Luna Baruah, Michaela Wenzel

**Affiliations:** Division of Chemical Biology, Department of Biology and Biological Engineering, Chalmers University of Technology, 412 96 Gothenburg, Sweden; sidarta@chalmers.se (M.S.); baruah@chalmers.se (L.B.)

**Keywords:** mechanosensitive channels, osmotic stress, osmotic down-shock, hypoosmotic stress, antibiotics, antimicrobial peptides, bacterial-stress response

## Abstract

Bacteria accumulate osmolytes to prevent cell dehydration during hyperosmotic stress. A sudden change to a hypotonic environment leads to a rapid water influx, causing swelling of the protoplast. To prevent cell lysis through osmotic bursting, mechanosensitive channels detect changes in turgor pressure and act as emergency-release valves for the ions and osmolytes, restoring the osmotic balance. This adaptation mechanism is well-characterized with respect to the osmotic challenges bacteria face in environments such as soil or an aquatic habitat. However, mechanosensitive channels also play a role during infection, e.g., during host colonization or release into environmental reservoirs. Moreover, recent studies have proposed roles for mechanosensitive channels as determinants of antibiotic susceptibility. Interestingly, some studies suggest that they serve as entry gates for antimicrobials into cells, enhancing antibiotic efficiency, while others propose that they play a role in antibiotic-stress adaptation, reducing susceptibility to certain antimicrobials. These findings suggest different facets regarding the relevance of mechanosensitive channels during infection and antibiotic exposure as well as illustrate that they may be interesting targets for antibacterial chemotherapy. Here, we summarize the recent findings on the relevance of mechanosensitive channels for bacterial infections, including transitioning between host and environment, virulence, and susceptibility to antimicrobials, and discuss their potential as antibacterial drug targets.

## 1. Introduction

**Figure 1:**
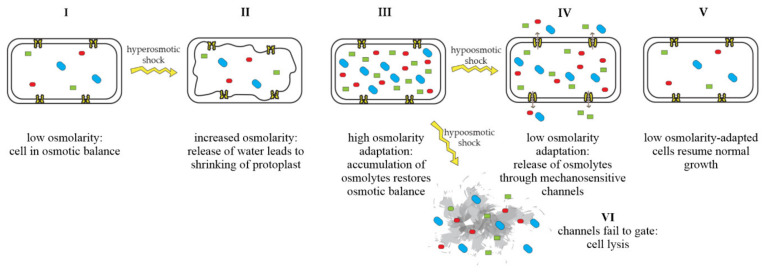
Adaptation of bacterial cells to changing osmotic pressure. A sudden increase in osmolarity leads to loss of water and shrinking of the protoplast, counteracted by the accumulation of compatible solutes, which raise intracellular-solute concentrations and restore the cell’s osmotic balance (**I**–**III**). A sudden decrease in osmolarity leads to an influx of water, resulting in increased turgor pressure, which is relieved by the opening of mechanosensitive channels and subsequent release of osmolytes into the environment (**IV**), restoring osmotic balance (**V**). If mechanosensitive channels fail to gate, cells lyse (**VI**). Figure adapted from Booth et al. [22].

Mechanosensitive channels are integral membrane proteins that are present in the membranes of bacteria, archaea, and eukaryotes [1,2]. These channels are closed under normal conditions and open in response to mechanical-membrane stretch [3]. This mechanosensitive response plays a role in a variety of biological functions such as hearing, touch, and cardiovascular regulation [4]. In bacteria, the role of mechanosensitive channels is that of an emergency-release valve. In response to a sudden shift from high to low osmolarity, as, e.g., experienced by soil bacteria after heavy rainfall, which leads to an influx of water, mechanosensitive channels release osmolytes to relieve increased turgor pressure and prevent cells from bursting (Figure 1) [5,6].

Osmolytes, also referred to as osmoprotectants, are highly soluble organic compounds that do not interfere with cellular processes. Amino acids and derivatives (glutamate, glutamine, aspartate, proline, betaines), sugars (sucrose, trehalose), polyols (glycerol, arabitol, inositol), and many other compounds can assume the role of osmoprotectants [7,8,9]. In response to hypotonic challenges, Bacterial Mechanosensitive channels have been shown to release osmolytes, such as glycine betaine, glutamate, and trehalose [10,11,12].

Bacterial Mechanosensitive channels are often viewed from an environmental or biotechnological perspective, e.g., enhanced glutamate secretion in the industrial amino-acid producer *Corynebacterium glutamicum*. Yet, they have also been reported to play a role in bacterial pathogenesis [13,14,15,16,17] and appear to be of importance for the susceptibility of different bacterial species to antimicrobials [18,19,20]. Interestingly, some studies have shown that mechanosensitive channels increase the potency of certain antibiotics, by serving as their entry gates into bacterial cells [19,20], while others have observed that bacteria adapt to antibiotic-induced membrane stress by opening mechanosensitive channels that contribute to antibiotic tolerance [11,21]. In this review, we summarize recent developments on the role of Bacterial Mechanosensitive channels regarding antibiotic susceptibility and pathogenesis.

## 2. Structure and Gating of Bacterial Mechanosensitive Channels

A bacterial cell typically harbors multiple mechanosensitive channels with different properties that allow a nuanced temporal response to various levels of hypoosmotic stress. These different properties are related to conductance, gating kinetics, sensitivity to membrane tension, and structure of the channel proteins. Based on these properties, mechanosensitive channels are typically divided into three classes: MscL (mechanosensitive channel of large conductance), MscS (mechanosensitive channel of small conductance), and MscM (mechanosensitive channel of mini conductance) [3,23]. However, in *Escherichia coli*, an additional class of mechanosensitive channel has been described. This class, named MscK (mechanosensitive channel K^+^), is structurally similar to MscS, but its gating requires lower membrane tension and high extracellular concentrations of potassium [24].

Both MscL- and MscS-type channels have been studied extensively, and crystal structures are available [25,26]. MscL-family proteins are moderately to highly conserved, whereas MscS-family proteins exhibit a broader structural diversity [2]. Typically, microorganisms possess only one MscL but multiple MscS homologs [27].

There are two model organisms that have been used predominantly to study Bacterial Mechanosensitive channels, the standard Gram-negative model *E. coli* and the ubiquitous Gram-positive soil bacterium *Bacillus subtilis*. However, *C. glutamicum* and its relative *Mycobacterium tuberculosis* have also been used as models to examine the structural and functional aspects of mechanosensitive channels. 

A plethora of studies have been performed on these channels and many excellent reviews are available on their structure, gating mechanisms, and function in osmoadaptation [3,6,23,27,28,29,30,31,32]. Therefore, we will not address these points in detail, but briefly outline the key aspects of their structure and gating mechanisms that are relevant to understand their role in antimicrobial resistance and pathogenesis, which shall be the main focus of this review.

### 2.1. Structural Determinant of MscL and MscS Gating

**Figure 2:**
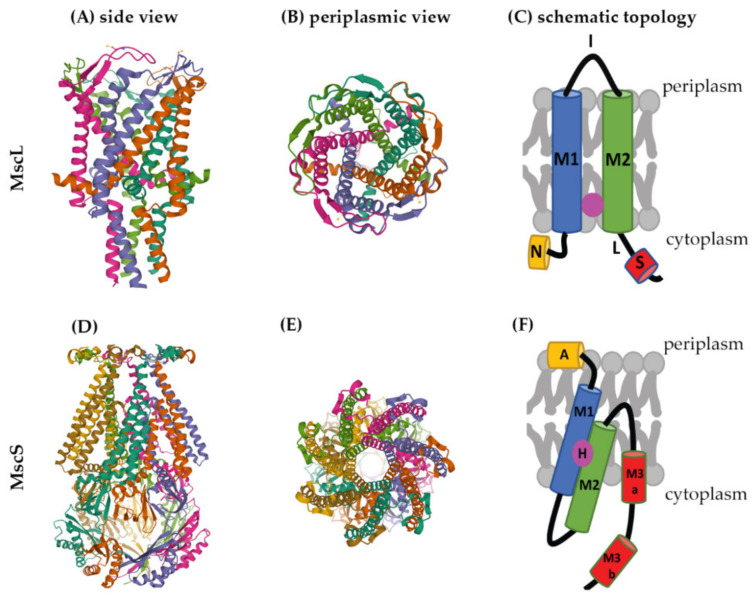
Structures of MscL and MscS. The structure of Mt-MscL (**A**–**C**) was derived from X-ray crystallography (PDB: 2OAR) [33]. (**A**) Side view, (**B**) periplasmic view, (**C**) schematic topological depiction of an MscL monomer. The structure of Ec-MscS (**D**–**F**) was derived from cryo-electron microscopy in lipid nanodiscs (PDB: 6PWP) [34]. (**D**) Side view, (**E**) periplasmic view, (**F**) schematic topological depictions of an MscS monomer. Schematic topological depictions of MscL and MscS were adapted from Pivetti et al. and Reddy et al. [1,34], respectively (relative sizes of domains not to scale). A: Anchor, N: N-terminal α-helix, M1, M2, M3a, and M3b: transmembrane domains, I: periplasmic loop, L: linker, S: short C-terminal region, H: hook lipid.

Several crystal structures are available for mechanosensitive channels (Supplementary Table S1). Two of these have become major models to study the structures and molecular-gating mechanisms of mechanosensitive channels, *M. tuberculosis* MscL (Mt-MscL, PDB: 2OAR) and *E. coli* MscS (Ec-MscS, PDB: 6PWP) (Figure 2) [33,34].

MscL channels form homopentamers, as shown for Mt-MscL in Figure 2A,B [33]. Each subunit of this pentamer has an N-terminal α-helix along the membrane (N), two α-helical transmembrane domains (M1 and M2), a periplasmic loop (I) that connects M1 and M2, and a short C-terminal region (S) linked to M2 via a flexible linker (L) (Figure 2C) [1,25,33,35].

M1 and M2 form the transmembrane channel, while the C-terminal region forms a cytoplasmic bundle below the channel pore and has been proposed to maintain the closed state of the channel [1,22]. The N-terminus of MscL acts as a “slide helix” that runs along the cytoplasmic membrane and stabilizes the M1 domain [35]. 

Recently, a hydrophobic nanopocket in the transmembrane region of MscL, situated between M1 and M2, sitting close to the surface of the inner membrane leaflet, has been identified and proposed to be essential for channel gating [36,37]. In the current model, this nanopocket is in contact with membrane-lipid fatty-acid chains, which act as negative modulators and prevent channel gating. Removal of lipid chains from the nanopocket through a membrane stretch is proposed to activate MscL [36]. 

MscS is a homoheptamer with a large cytoplasmic domain (Figure 2D,E) [3,38,39]. This cytoplasmic domain is believed to act as a molecular sieve that balances the passage of positive and negative osmolytes as well as ensures a net-neutral efflux in order to conserve the transmembrane potential [40]. The cytoplasmic domain has also been suggested to be a sensor for the excessive cytoplasmic crowding involved in preventing cytoplasm over-draining [41].

**Figure 3:**
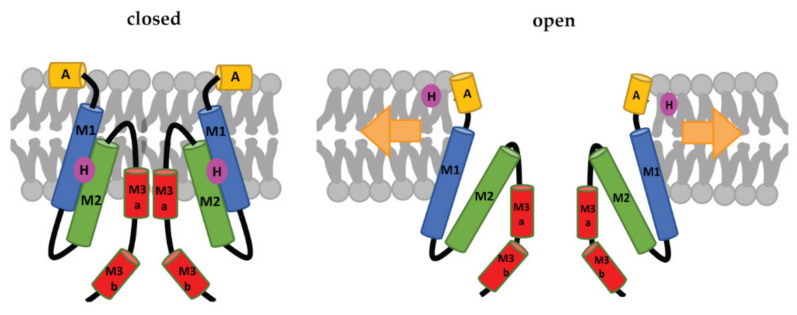
Schematic representation of the MscS-gating mechanism proposed by Reddy et al. [34]. Arrows represent ‘force-from-lipids’. The relative sizes of the domains are not to scale. A: Anchor, M1, M2, M3a, and M3b: transmembrane domains, H: hook lipid. The figure shows a model, in which the hook lipid moves out of its binding pocket upon membrane stretch and corresponding channel opening. An alternative model, where the hook lipid stays situated between M1 and M2, was not yet ruled out [34].

Each subunit of the MscS heptamer has three membrane-spanning helices: M1, M2, and M3 (Figure 2D–F). M1 and M2 form a sensor for membrane tension. M3 consists of two parts: the hydrophobic M3a that lines the pore and the amphipathic M3b situated at the membrane-cytoplasm interface [26]. An N-terminal domain, called Anchor (A), has recently been identified by cryo-electron microscopy (Figure 2F). This anchor is situated at the outer-leaflet interface and was shown to be essential for channel gating [34]. The new cryo-electron-microscopy structure also revealed the presence of a ‘hook’ lipid (H) that hooks to the top of each M2–M3 hairpin. This hook lipid is believed to facilitate force transition from the lipid bilayer to the transmembrane domains of the channel (Figure 3). However, it is yet unclear whether the hook lipid is removed from its binding pocket during membrane stretch or not [34].

### 2.2. Importance of Membrane Lipids for the Gating Mechanism

The paradigm of the “force-from-lipid” model, in which, in the simplest of terms, a membrane stretch pulls the channel apart to open, has been long established [42,43]. In this model, the lipid bilayer influences channel gating by directly interacting with mechanosensitive channels or by modulating the global properties of the cell membrane, such as membrane thickness, membrane fluidity, membrane curvature, hydrophobic interactions, and many more [42,44,45].

In recent years, the roles of specific lipids and lipid–protein interaction sites, such as the hydrophobic lipid chain-binding pocket of MscL and the hook lipid binding to MscS, have been revealed. A recent cryo-electron-microscopy study has examined Ec-MscS in different membrane environments, mimicking stretched and unstretched conditions as well as supporting the diverse and complex roles of lipids in the gating of MscS [46]. This includes pore lipids, which prevent the passage of molecules through the channel in the closed state, gatekeeper lipids that stabilize the closed conformation of the channel and dissociate during membrane stretch, and pocket lipids that sit in pockets between subunits and are pulled out under membrane tension, transferring the force of the membrane stretch to the channel protein [46].

Not only can specific membrane lipids in the immediate proximity of the channels play a role in gating but also the overall membrane composition and organization critically influence the behavior of mechanosensitive channels. Since they respond to membrane stretch, factors such as overall membrane thickness and fluidity are the first to come to mind. Water influx into bacterial cells during hypoosmotic shock will increase the turgor pressure against the cell wall. This will stretch the bilayer, concomitantly leading to membrane thinning, fluidization, and increased membrane tension [47].

In fact, Ec-MscL was reported to be sensitive to lipid-bilayer thickness in vitro [48,49]. Thus, a decrease in bilayer thickness led to a lowered gating threshold for MscL, whereas an increase in bilayer thickness led to an increased MscL-gating threshold [49].

In contrast, Ec-MscS was reported to be less sensitive to bilayer thickness [49], indicating a different responsiveness from MscL and MscS. This was supported by another study, which showed that altering fatty-acyl chain length did not notably alter Ec-MscS-gating thresholds [45]. However, a significant shift in tension sensitivity was observed, when more rigid model membranes were used, suggesting that a less-fluid membrane environment, e.g., through enrichment of fully saturated lipids, hampers Ec-MscS gating [45].

Similarly, surface-adhesion forces have been observed to trigger mechanosensitive-channel opening in biofilms of *Staphylococcus aureus* [50]. Channel gating increased with increased adhesion force. It is believed that, when bacteria attach to a surface during the first stage of biofilm formation, adhesion forces will deform the bacterial cell wall and, consequently, generate sufficient membrane tension to trigger channel opening [51,52]. It is likely that adhesion forces work in concert with other driving forces, such as thickness and fluidity, to modulate channel gating.

## 3. Role of Mechanosensitive Channels during Infection

While new discoveries are being made regularly about the structural determinants of mechanosensitive-channel gating, their biological role as emergency-release valves that protect bacteria during hypoosmotic shock is long-standing and well-established. Thereby, they are typically viewed in the context of environmental challenges, e.g., the adaptation of soil bacteria such as *B. subtilis* to heavy rainfalls. However, in recent years, more and more evidence has been discovered about how they also play an important role for pathogenic bacteria during infection. For example, mechanosensitive channels play a role in adapting to osmotic changes occurring upon transitioning from the environment to the host and back [13,16]. They may also play an important role in adapting to changing osmotic conditions within the body, e.g., in bladder infections, where osmolarity can change dramatically depending on the patient’s water intake [53]. In the following, we will review the importance of mechanosensitive channels for transitioning between environment and host, host colonization, and virulence.

### 3.1. Transition between Host and Environment

Upon transitioning from the environment to the host (and vice versa), pathogens suddenly face a drastic osmotic change and need suitable adaptation mechanisms to survive this challenge. Typically, the human or animal body will constitute a higher osmolarity environment than many natural reservoirs, such as freshwater. For example, *Francisella tularensis*, a pathogen that causes tularemia disease in mammals [54,55,56], needs an MscS-like channel (Ft-MscS) to survive the transition from its mammalian host to freshwater [16]. Similarly, *Campylobacter jejunii*, a major cause of bacterial gastroenteritis in humans [57], needs MscS homologs to survive the hypoosmotic stress that occurs during transmission from the digestive tract of the host to the environment [13]. In both cases, mechanosensitive channels are necessary to maintain natural bacterial reservoirs and, thus, play a role in the environmental persistence and transmission of these pathogens. *F. tularensis* has been known to cause waterborne-tularemia outbreaks in several countries [54,55,56], and *Campylobacter* spp. are on the WHO’s list of antibiotic-resistant bacteria, against which new antibiotic treatments are most urgently needed [58]. Thus, understanding the role of mechanosensitive channels in transitioning from host to environment and their relevance for maintaining natural reservoirs, allowing transmission and spread of these bacteria, is of high relevance.

In some cases, mechanosensitive channels play a role in transitioning from the environment to the host and enable bacteria to colonize host tissues [14,15,17]. One example for this is *Salmonella typhimurium*. Studies have shown that the mechanosensitive channel YnaI is required for host-intestinal colonization, and the deletion of the *ynaI* gene in *S. typhimurium* leads to an increased internalization in macrophages [14,17]. Another notable example is *Neisseria gonorrhoeae*, which causes gonorrhea by colonizing the mucosal epithelia of the human urogenital tract. During infection, *N. gonorrhoeae* may experience fluctuating osmotic conditions, e.g., during the passing of urine. An MscS-like channel (Ng-MscS) has been shown to be essential for osmotic downshock survival in this organism, and the *N. gonorrhoeae* wild type outcompeted a mutant lacking this channel, with respect to colonization and survival, in a murine vaginal-tract-infection model, putting forward the importance of Ng-MscS in host colonization [15]. 

### 3.2. The Urinary Tract as an Osmotically Challenging Environment in the Human Body

The urinary tract is the most prominent example of an environment with drastically fluctuating osmolarity within the human body. Generally, urine is a complex, hypertonic medium with low pH as well as high salt and urea content [59]. In healthy adults, urine typically contains glucose (0.2–0.6 mM), creatine (0.38–55.6 mM), citrate (1.0–2.0 mM), sucrose (70–200 µM), manganese, amino acids, and traces of fatty acids [59]. However, depending on factors such as diet, water intake, frequency of passing urine, and a number of health conditions, the osmolarity of urine can vary considerably. For example, kidney urine usually has higher osmolarity and lower pH compared to bladder urine [59]. This variability poses dramatic osmotic challenges to bacteria colonizing the bladder and urethra. Despite being a generally harsh and challenging environment, uropathogens can survive and even thrive in the urogenital tract. These pathogens utilize urine contents as nutrients and rely on their osmoadaptive mechanisms to survive the stressful osmotic conditions in this environment [59]. 

Several studies have demonstrated the importance of osmoadaptation mechanisms for uropathogens, yet this has mostly been studied for high-osmolarity-adaptation strategies. For example, Culham et al. have shown that the osmoregulatory proline-transporter ProP has higher activity in the *E. coli* pyelonephritis isolate HU734 compared to the *E. coli* lab strain K-12 [53]. Deletion of *proP* impaired the in vitro growth of *E. coli* HU734 in human urine but not in a high-osmolarity minimal medium. Moreover, the deletion of ProP also reduced bladder colonization by HU734 [53]. Furthermore, it has been shown that the survival of uropathogenic *E. coli* in the urinary tract depends on OmpR, which is a part of the EnvZ-OmpR regulatory system that responds to hyperosmotic stress [60]. 

The osmolarity of urine has been suggested to influence the virulence of uropathogens [61,62]. Thus, the production of virulence factors in *Pseudomonas aeruginosa* increased when osmolarity was raised from 200 to 300 mOsmol/L [62]. However, a significant decrease in both growth and production of virulence factors was observed with a further increase in osmolarity. Additionally, *P. aeruginosa* grown in high osmolarity-medium (300 mOsmol/L) was more resistant to phagocytosis and more virulent in a mouse model than the same strain grown in nutrient broth.

These studies show that osmoadaptive mechanisms are crucial for the growth of uropathogens and colonization in the urinary tract. While the importance of hyperosmotic-stress-adaptation strategies in urine is rather clear, virtually nothing is known about the importance of low-osmolarity adaptation measures such as mechanosensitive channels. Yet, considering that the osmolarity of urine can decrease rapidly and considerably, e.g., by imbibing a large amount of water, it is reasonable to assume that mechanosensitive channels may play a role in this environment as well. This notion is supported by the apparent importance of Ng-MscS in *N. gonorrhoeae* colonization of the urogenital tract [15] and will be an interesting subject for future research.

## 4. Impact on Antibiotic Susceptibility

**Figure 4:**
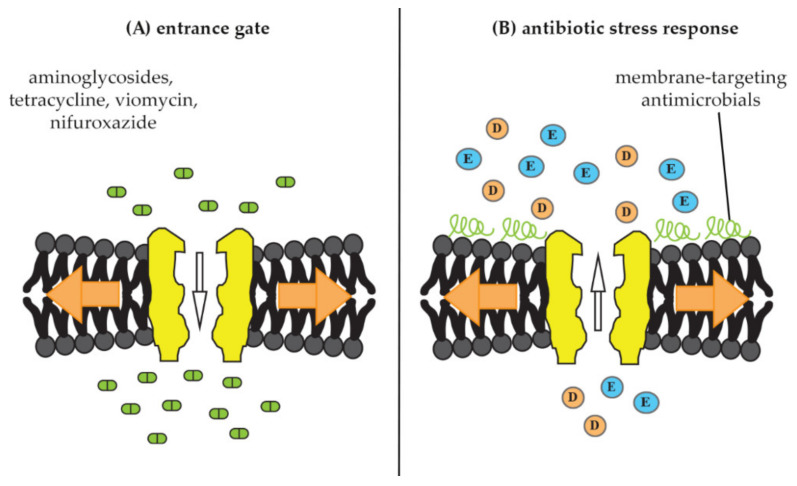
Documented interactions of mechanosensitive channels with antimicrobials. (**A**) Mechanosensitive channels as entrance gate for antimicrobials into bacterial cells. Upon membrane stretch (orange arrows), channels open and allow better uptake of certain antibiotic molecules, such as the aminoglycosides dihydrostreptomycin, spectinomycin, and kanamycin as well as tetracycline, viomycin, and nifuroxazide [19,20]. (**B**) Mechanosensitive channels as part of an antibiotic-stress response. Treatment with membrane-active antimicrobials, such as MP196, nisin, gramicidins, and aureins mimics membrane stretch and triggers release of osmoprotective amino acids (E = glutamate, D = aspartate), resulting in osmotic-membrane stabilization and decreased antibiotic susceptibility [11,21].

In addition to their possible roles in virulence and pathogenesis, mechanosensitive channels have also been shown to impact the activity of antibiotics. Thus, studies have shown that they may serve as entrance gates for certain antibiotic classes into bacterial cells (Figure 4A) [11,19,20]. Conversely, other antimicrobial compounds appear to be able to trigger mechanosensitive-channel opening and it has been suggested that they play a role in stress adaptation and resistance towards such compounds [11] (Figure 4B). In the following, we will review the importance of mechanosensitive channels for antibiotic sensitivity.

### 4.1. Mechanosensitive Channels as Antibiotic Entry Point

Several studies have shown that the potency of some antibiotics depends on or increases with the presence of mechanosensitive channels (Table 1) [18,19,20]. This has been explained by these channels acting as an entrance route for these antimicrobials to bacterial cells [19,20]. This was, for example, the case for the aminoglycoside dihydrostreptomycin, which can bind to and change the conformation of Ec-MscL, allowing the efflux of glutamate and potassium into the environment and the influx of the antibiotic itself into the cell [19,20]. Spectinomycin, another aminoglycoside, exhibited a similar dependency on Ec-MscL [19]. These findings suggest that using MscL as entry point is not an exclusive feature of streptomycin but a common property of aminoglycosides.

Supporting this notion, another study demonstrated that hypoionic shock specifically enhances the potential of aminoglycosides to eradicate bacterial persisters. Strikingly, mechanosensitive-channel activators, such as indole or parabens, greatly increased this effect [71]. Furthermore, rapid freezing has been shown to enhance the bactericidal activity of aminoglycosides against pathogenic bacteria, including persisters. This was explained by cell-membrane destabilization resulting in the activation of MscL, which subsequently enhanced the uptake of aminoglycosides into the cells [72]. This phenomenon was not observed for β-lactams and fluoroquinolones, so it was suggested that it is specific for aminoglycosides.

However, other compounds have by now also been shown to enter bacterial cells through mechanosensitive channels. Thus, tuberactinomycin viomycin and nitrofuran nifuroxazide turned out to be dependent on both Ec-MscS and Ec-MscL [19]. Similarly, curcumin, an antibacterial compound found in tumeric, was recently reported to have activity dependent on MscL (but not on MscS) [63]. Curcumin has been shown to be able to activate Ec-MscL, both by patch-clamp analysis of native-bacterial membranes and in vivo physiology/flux studies, indicating that MscL also serves as an entrance pathway for curcumin [63]. Moreover, recent studies on MscL-specific agonists suggested that tetracycline also exhibits an MscL-dependent activity and might use this channel as an entry point [64,65]. Further studies will be needed to assess how widespread this mechanism truly is.

MscL-dependent antibacterial activity has also been observed for sublancin 168, a lantibiotic with an as-yet-unknown mechanism of action [18]. Susceptibility to sublancin 168 was observed to depend on osmolarity, as high NaCl concentrations reduced the sensitivity of both *B. subtilis* and *S. aureus*. While it is common for antimicrobials, especially antimicrobial peptides, to exhibit a salt-dependent activity, the authors could show that NaCl did not influence the production, activity, or stability of sublancin 168 itself. Furthermore, deletion of the *mscL* genes rendered a sensitive strain resistant against the lantibiotic, regardless of the presence of NaCl [18]. However, it has not yet been resolved whether MscL serves as the entry point for sublancin 168, constitutes a target, or is involved in another process that promotes the bactericidal activity of this lantibiotic.

### 4.2. Mechanosensitive-Channel Activation as Antibiotic-Stress Response

In contrast to these examples, where mechanosensitive channels enhanced the activity of antibiotics, other studies have found that they may also act as part of an antibiotic-stress response, which protects bacterial cells from membrane-targeting antibiotics [11,21]. This was first discovered for the antimicrobial peptide MP196, which disturbs membrane architecture leading to the displacement of peripheral-membrane proteins. Additionally, this peptide induced the release of glutamate and aspartate from *B. subtilis* cells into the culture medium, a response that was also observed with osmotic downshock. Importantly, this release was specific for these amino acids, not caused by pore formation, and could be impaired by deleting the four mechanosensitive channels in this organism [11]. The quadruple-mechanosensitive-channel mutant was also more sensitive to MP196. Importantly, supplementation of the culture medium, with exogenous glutamate strongly decreasing the susceptibility of *B. subtilis* for MP196. A similar effect was seen with NaCl and KCl, suggesting that this effect was due to osmostabilization [11]. 

A similar glutamate/aspartate release was also observed in response to other membrane-targeting antimicrobial peptides, including gramicidin A, gramicidin S, nisin, and aureins 2.2, 2.3, and 2.2Δ3 [11,21]. Thus, mechanosensitive channels appear to be involved in a protective-stress response to a range of membrane-active antimicrobials.

Resembling these observations, the industrial amino-acid producer *C. glutamicum* excretes glutamate through its mechanosensitive channels MscCG (NCgl1221) and MscCG2, in response to penicillin [67,68]. Deleting both *mscCG* and *mscCG2* strongly decreases the excretion of glutamate, which is restored by complementation of either one of them. Interestingly, heterologous expression of Ec-MscS in the *C. glutamicum mscCG* deletion strain also restores ampicillin-induced glutamate excretion [66].

As reviewed by Nakayama et al. [28], *C. glutamicum* only overproduces glutamate when the cell envelope is compromised by specific treatments, such as biotin limitation, the addition of fatty-acid ester surfactants (tween 40 and tween 60), or antibiotics that inhibit cell-wall synthesis. It has been suggested that these treatments increase membrane tension and weaken the cell wall, resulting in osmotic pressure allowing glutamate efflux [28].

Apart from L-glutamate, MscCG has also been proposed to transport L-aspartate and L-phenylalanine [73]. Thus, an *mscCG*-deletion strain grown in biotin-depleted medium accumulated higher intracellular L-glutamate and L-aspartate pools than the wild type, suggesting that these channels transport both of these molecules [73]. A patch-clamp analysis of a *B. subtilis* strain, which was heterologously expressing MscCG, suggested that both glutamate and aspartate were exported through MscCG by passive diffusion [74]. Both studies found a preference of MscCG for glutamate over aspartate. While corresponding studies on the *B. subtilis* proteins are lacking, it was likewise observed that antimicrobial peptide-treated cells released more glutamate than aspartate [11,21], suggesting that a similar preference may exist for the *B. subtilis* channels as well.

Interestingly, penicillin-induced glutamate production in *C. glutamicum* led to the upregulation of genes encoding MscCG as well as genes involved in cellular-defense mechanisms [75]. Hirasawa et al. suggested that these genes might be transcriptionally activated as part of a penicillin-stress response. Yet, their connection with glutamate production and excretion remains unclear. 

Similarly, ampicillin has been shown to induce the transcription of *mscL* and *mscS* in *E. coli* [69,70]. This induction was not observed with other antibiotics, such as norfloxacin, gentamicin, and ofloxacin, which is in line with cell-envelope-compromising conditions triggering channel opening. Overexpression of MscS protects cells against sub-inhibitory ampicillin concentrations. Interestingly, this effect is diminished by mutating its cytoplasmic domain [76]. Biochemical screens, aimed at measuring the effects of diverse metabolite supplementations on antibiotic susceptibility, have revealed that L-ornithine, L-arginine, and D-glutamate supplementation reduce the activity of ampicillin [77]. This is similar to decreased susceptibility of *B. subtilis* to MP196, upon supplementation with glutamate [11]. 

In several of these studies it was hypothesized that the respective antibiotics and stress conditions trigger mechanosensitive-channel opening, by mimicking a membrane stretch similar to that caused by increased turgor pressure. This hypothesis would explain why only cell-envelope-compromising conditions caused the observed effects. Yet so far, the molecular mechanisms underlying antibiotic-induced mechanosensitive-channel gating and amino-acid release remain elusive.

### 4.3. Osmolarity and Host-Defense Peptides

The observation that not only glutamate/aspartate release but also exogenous supplementation of glutamate and salt appears to protect bacterial cells from membrane-active antibiotics, which sheds new light on the salt-sensitivity of antimicrobial peptides [11,21]. It is well-established that the activities of many antimicrobial peptides are significantly reduced under high-salinity conditions, which is often explained by either blocking of negatively charged binding sites on the cell membrane by cations or by electrostatic interactions of salt with the peptides themselves [78,79,80,81]. However, the aforementioned studies suggest that salt may indeed provide an osmotic stabilization of the cell membrane, which elicits a protective effect against the action of membrane and cell-wall-active antibiotics. While the true impact of this possibility is yet to be ruled out, it is exciting to discuss an impact of this effect under infection conditions, e.g., on host-defense peptides. 

Few observations, particularly with respect to urogenital infections, may be related to such an osmostabilization effect. For example, both the killing and phagocytosis of *E. coli* and *Staphylococcus saprophyticus* by neutrophils are significantly impaired in urine, with higher osmolarity and lower pH [61]. Neutrophils contain host-defense peptides that are released in the phagosome upon pathogen ingestion [82]. It is tempting to speculate that their activity may be hampered in a high-osmolarity environment, as previously observed in vitro [11,21]. Asogwa et al. could show that an *S. typhimurium* strain, lacking its mechanosensitive channel YnaI, is more vulnerable to macrophages [17]. Possibly, this channel could play a role in a similarly protective-stress response as the aforementioned *B. subtilis*, *E. coli*, and *C. glutamicum* mechanosensitive channels. Moreover, several studies have shown that mechanosensitive channels are required for host colonization or virulence [11,14,15,17], so it is tempting to speculate that they do not only help in transitioning between environments of different osmolarity but may also protect against host-defense mechanisms such as antimicrobial peptides.

## 5. Mechanosensitive Channels as Novel Antimicrobial-Drug Targets

Whether mechanosensitive channels promote bacterial virulence and host colonization, serve as entry points for certain antibiotic classes into bacterial cells, or are part of a protective antibiotic-stress response, all these functions support the notion that these channels play an important role during infection, meaning they can possibly be exploited as a novel-drug target. Even though mechanosensitive channels are normally not essential in bacteria and their inhibition may not even cause growth defects under lab conditions, they may be essential for virulence or pathogenesis or may serve as an excellent target for antibiotic potentiators, either by promoting antibiotic uptake or by inhibiting bacterial-defense systems. They may even serve as potentiators for host immunity, by boosting antimicrobial-peptide activity. Furthermore, the modulation of channel gating into an ‘always open’ state is in fact very likely to be lethal due to unhindered intracellular-content leakage. Thus, mechanosensitive channels constitute rather versatile drug targets.

Mechanosensitive channels are conserved and structurally distinct from their mammalian counterparts [1,4], suggesting that selective inhibitors can be developed. Importantly, several compounds are already known that inhibit or modulate mechanosensitive-channel activity. While none of them has made it to a clinical study yet, they do demonstrate the druggability of these channels. So far, little effort has been put into developing mechanosensitive-channel inhibitors as antimicrobials or antibiotic potentiators, yet they do constitute an attractive new drug target to be explored more thoroughly in the future. Table 2 shows an overview of currently known inhibitors and modulators of mechanosensitive-channel activity, which will be discussed in the following.

### 5.1. Compounds Directly Targeting Mechanosensitive Channels

Few compounds have been developed that interact with and impair the function of MscL. Its strong conservation among bacteria and its structural distinction from mammalian mechanosensitive channels makes MscL an attractive target for drug development [35]. MscL lacks a selectivity filter, so constitutive gating of this channel will be detrimental for the cell, since its large pore will unselectively permeabilize the cell membrane, resulting in dissipation of the membrane potential and, consequently, cell death.

One of the first success stories in designing an MscL-targeting antibiotic compound is that of ramizol (previously ‘compound 10’) [88]. Ramizol was discovered in an in-silico screening approach and was predicted to interact with MscL. Indeed, the compound was shown to inhibit the growth of MscL-expressing *S. aureus* cells in vivo. Although less effective, ramizol also inhibited the growth of MscS-expressing cells, suggesting a nonspecific activation of mechanosensitive-channel gating in vivo. Moreover, it is likely that mechanosensitive channels may not be the only targets of ramizol: cells not expressing either MscL or MscS were, in fact, also inhibited when treated with higher concentrations of the compound, suggesting an additional, concentration-dependent mechanism of action. However, patch-clamp analyses revealed that ramizol only significantly reduced the gating threshold of MscL, indicating that it has at least a preference for this specific channel. Although its mechanism of action is not yet fully understood, ramizol has been shown to be effective in a *Caenorhabditis elegans* model of methicillin-resistant *S. aureus* infection, demonstrating its potential to be developed as a new therapeutic against antibiotic-resistant bacterial infections. Subsequently, ramizol has been further explored in pre-clinical studies; with respect to its efficacy against *Clostridium difficile* infections, its pharmacokinetics profile, dosage, and drug-delivery options [83,84,85,86,87] all show promising results.

In a different set of studies, two MscL-specific agonists, 011A and K05, have been evaluated for their potential as novel antibiotics [64,65,89]. These compounds bind to MscL and increase its sensitivity to membrane tension. Upon treatment with these compounds, improper gating of MscL led to the decreased viability of *E. coli* cells [65,89]. Cells lacking MscL were resistant to these compounds. Similar effects have been observed in other bacteria, such as *S. aureus* and *M. smegmatis* [64,65]. It has been demonstrated that a lysine residue at position 97 of Ec-MscL is the crucial binding site for both compounds [65,89]. When Bs-MscL, which lacks a lysine residue at the equivalent position, was heterologously expressed in an *E. coli mscL*-null mutant, the strain was insensitive to both compounds. Changing the corresponding Bs-MscL residue to lysine rendered it sensitive to both agonists.

Both A011 and K05 have been reported to increase the potency of dihydrostreptomycin, kanamycin, tetracycline, and ampicillin against both *S. aureus* and *M. smegmatis* [64,65], suggesting their potential use as antibiotic adjuvants. The authors propose that these compounds specifically permeabilize the membrane, by modulating MscL gating, and, thus, facilitate the uptake of antibiotics into the cytoplasm. While this may explain the increased activity of antibiotics with cytoplasmic targets (dihydrostreptomycin, kanamycin, tetracycline), this mechanism would not explain the increased potency of ampicillin, with a target that is located on the outside surface of the cell membrane. Since penicillin activates the mechanosensitive channels in *C. glutamicum* [28], and ampicillin induces their expression in *E. coli* [69,70], it can be speculated that the observed activity increase could be due to a different combined effect of the agonists with ampicillin. 

Very recently, a structurally novel class of MscL agonists that targets a similar binding pocket as A011 and K05, situated at the cytoplasm-membrane interface, has been discovered by in silico docking studies [90]. While the potential for clinical development of such compounds remains to be evaluated, these studies demonstrate the specific druggability of MscL.

### 5.2. Compounds Indirectly Modulating Channel Gating

In addition to molecules that directly interact with mechanosensitive channels, several compounds are known that modulate channel gating through an interaction with the lipid bilayer. For example, it has been shown that amphipathic molecules can activate mechanosensitive channels in giant *E. coli* spheroplasts, whereby their effectiveness is proportional to their hydrophobicity [100]. This is, for example, the case for parabens, which are amphipathic compounds that have been used as antimicrobials in the food and cosmetic industries [92]. They have been shown to spontaneously activate MscL and MscS [91]. Interestingly, parabens affected the sensitivity of MscS differently, depending on whether they were applied to the cytoplasmic or periplasmic side of excised membrane patches [92]. When added to the periplasmic side, parabens increased the sensitivity of MscS, while the opposite was the case when parabens were added to the cytoplasmic side of the membrane patch. It has been hypothesized that this effect may be due to the MscS gate being located in the cytoplasmic domain. When applied externally, parabens will insert into the outer leaflet, increasing tension in the inner leaflet and activating channel gating. In contrast, when applied internally, the insertion of parabens will increase the lateral pressure around the channel, creating a ‘squeezing’ effect, which hampers channel opening [92]. Although the antimicrobial mechanism of parabens remains unclear, it is unlikely that mechanosensitive channels are their primary target, as bacteria lacking these channels are still susceptible [92].

Another group of amphipathic molecules that affect mechanosensitive-channel gating are the piscidins (P1 and P3), which are histidine-enriched, alpha-helical antimicrobial peptides that have been shown to decrease the activating tension of MscS and MscL in *E. coli* spheroplasts [93,94]. Comert et al. suggest that piscidins may directly or indirectly modify the protein–lipid boundary, for example, by inducing membrane stretch or curvature, and re-direct forces acting on the lipid bilayer to the protein, resulting in a lower-activation threshold [93]. However, an *E. coli*-mutant lacking *mscL*, *mscS*, and *mscK*, does not markedly differ from the wild type in its sensitivity to piscidins, indicating that mechanosensitive channels are not their primary targets [94].

Another compound that is able to modulate mechanosensitive-channel activity is the spider venom GsMTx4, a globular amphipathic peptide. GsMTx4 has been reported to possess antimicrobial activity and is significantly more active against Gram-positive bacteria [97]. Intriguingly, patch-clamp experiments revealed a biphasic response of *E. coli* MscS and MscK, when the peptide was applied at the periplasmic side of the membrane patch: low peptide concentrations (2–4 µM) decreased the sensitivity of the channels to pressure, but the opposite was the case when higher peptide concentrations (>12 µM) were used [95]. In another study, it was shown that applying GsMTx4 to the cytoplasmic side increases the opening rate of MscL and MscS. This was attributed to the peptide binding to the lipid interface, locally increasing the membrane tension and, thereby, stabilizing the expanded conformation of the mechanosensitive channels [96].

In contrast to these varied activities of GsMTx4, gadolinium chloride (GdCl_3_) exclusively acts as an inhibitor that blocks mechanosensitive-channel opening [98,99]. Administration of 100 µM GdCl_3_ has been reported to completely abolish channel gating in the patch-clamp analyses of *E. coli*, *B. subtilis*, and *Enterococcus faecalis* mechanosensitive channels [98]. Another in vitro study demonstrated that GdCl_3_ could only inhibit MscL gating in the presence of anionic phospholipids, possibly because they mediate the interaction of the Gd^3+^ ion with the cell membrane [99]. Interaction of the gadolinium ions is assumed to induce lipid-bilayer compaction, resulting in increased lateral pressure that will “squeeze” the channels into their closed state. Mechanosensitive channels can be reactivated by washing out GdCl_3_ traces from the membrane [98,99]. 

While the potential of molecules that impact mechanosensitive channels through a disturbance of the lipid bilayer as antibacterial drugs is yet to be determined, which may possibly be hampered by specificity and selectivity issues, such compounds are at the very least helpful for studying the function of mechanosensitive channels and have already essentially contributed to their characterization [101,102,103,104,105,106]. So far, little effort has been put into developing such compounds as antibacterial drugs, yet with the continued antibiotic-resistance crisis, they may resurface as novel-drug candidates.

## 6. Conclusions

Bacterial Mechanosensitive channels have been extensively studied, with respect to their structures and gating mechanisms, yet their biological function is typically attributed to counteracting hypoosmotic challenges, mostly in environmental settings. In this review, we have highlighted studies that shed light on their functions with respect to infection scenarios. While not extensively studied yet, several studies have shown or suggested an important role for mechanosensitive channels in the transitioning of pathogens between host and environment (and vice versa), in host colonization, and in virulence. It is reasonable to assume that they may play a role in infection sites with fluctuating osmolarity, such as the urogenital tract. Future studies are needed to shed light on the function and importance of these channels in such infection scenarios. Furthermore, mechanosensitive channels have been implicated as entry points for antibiotics into cells, as antibiotic-stress response systems, and as targets for potential future antibiotics and potentiators. So far, we have just scraped the surface of the apparently diverse connections of mechanosensitive channels to antimicrobial chemotherapy and have not yet explored their potential as novel-drug targets. Yet, the first steps have been made that clearly show the great potential for new groundbreaking discoveries in this area, so it will be very interesting to see where these leads will go.

## Figures and Tables

**Table 1:** Antimicrobials known to be affected by mechanosensitive-channel activity.

Compounds Entering Cells through Mechanosensitive Channels	Compounds Triggering Amino-Acid Release from Mechanosensitive Channels
dihydrostreptomycin [19,20]	MP196 [11]
spectinomycin [19]	gramicidin A [11]
viomycin [19]	gramicidin S [11]
nifuroxazide [19]	nisin [11]
curcumin [63]	aurein 2.2 [11,21]
tetracycline [64,65]	aurein 2.3 [21]
* sublancin 168 [18]	aurein 2.2Δ3 [21]
	* penicillin [28,66,67,68,69,70]
	* ampicillin [28,66,67,68,69,70]

* indirect evidence.

**Table 2:** List of known inhibitors and modulators of mechanosensitive-channel activity.

Compound	Target	Mechanism	Structural Class	Activity Shown against
*compounds directly targeting mechanosensitive channels*				
ramizol [83,84,85,86,87,88]	MscL MscS	reduces gating threshold	styrylbenzene	*S. aureus*, *Streptococcus pneumoniae*, *Clostridium difficile*
011A [64,65,89]	MscL	stabilizes open state, increases permeability for antibiotics	small organic molecule	*E. coli*, *S. aureus*, *Mycolicibacterium smegmatis*
K05 [64,65,89]	MscL	stabilizes open state, increases permeability for antibiotics	small organic molecule	*E. coli*, *S. aureus*, *M. smegmatis*
compound 262 [90]	MscL	stabilizes open state, increases permeability for antibiotics	small organic molecule	*E. coli*, *M. tuberculosis*
*compounds indirectly targeting mechanosensitive channels*				
parabens [91,92]	MscL MscS	modulates gating	4-hydroxylbenzoic acid ester	*E. coli*
piscidins (P1 and P3) [93,94]	MscL MscS	sensitizes channel gating	alpha-helical peptide	*E. coli*
GsMTx4 [95,96,97]	MscL MscS	promotes or inhibits channel gating; concentration-dependent	globular peptide	*E. coli*
gadolinium chloride (GdCl_3_) [98,99]	MscL MscS	inhibits channel gating	inorganic salt	*E. coli*, *B. subtilis*, *Enterococcus faecalis*

## Data Availability

Data sharing is not applicable.

## Supplementary Materials

The following supporting information can be downloaded at: https://www.mdpi.com/article/10.3390/ph15070770/s1, Table S1: Available PDB structures of mechanosensitive channels [33,34,46,107,108,109,110,111,112,113,114,115,116,117,118,119,120,121,122,123].

## References

[1] Pivetti C.D., Yen M.-R., Miller S., Busch W., Tseng Y.-H., Booth I.R., Saier M.H. (2003). Two Families of Mechanosensitive Channel Proteins. Microbiol. Mol. Biol. Rev..

[2] Booth I.R., Miller S., Müller A., Lehtovirta-Morley L. (2015). The Evolution of Bacterial Mechanosensitive Channels. Cell Calcium.

[3] Kung C., Martinac B., Sukharev S. (2010). Mechanosensitive Channels in Microbes. Annu. Rev. Microbiol..

[4] Árnadóttir J., Chalfie M. (2010). Eukaryotic Mechanosensitive Channels. Annu. Rev. Biophys..

[5] Levina N. (1999). Protection of *Escherichia coli* Cells against Extreme Turgor by Activation of MscS and MscL Mechanosensitive Channels: Identification of Genes Required for MscS Activity. EMBO J..

[6] Booth I.R., Blount P. (2012). The MscS and MscL Families of Mechanosensitive Channels Act as Microbial Emergency Release Valves. J. Bacteriol..

[7] Csonka L.N. (1989). Physiological and Genetic Responses of Bacteria to Osmotic Stress. Microbiol. Rev..

[8] Bougouffa S., Radovanovic A., Essack M., Bajic V.B. (2014). DEOP: A Database on Osmoprotectants and Associated Pathways. Database.

[9] Hoffmann T., Bremer E. (2011). Protection of *Bacillus subtilis* against Cold Stress via Compatible-Solute Acquisition. J. Bacteriol..

[10] Hoffmann T., Boiangiu C., Moses S., Bremer E. (2008). Responses of *Bacillus subtilis* to Hypotonic Challenges: Physiological Contributions of Mechanosensitive Channels to Cellular Survival. Appl. Environ. Microbiol..

[11] Wenzel M., Chiriac A.I., Otto A., Zweytick D., May C., Schumacher C., Gust R., Albada H.B., Penkova M., Krämer U. (2014). Small Cationic Antimicrobial Peptides Delocalize Peripheral Membrane Proteins. Proc. Natl. Acad. Sci. USA.

[12] Ajouz B., Berrier C., Garrigues A., Besnard M., Ghazi A. (1998). Release of Thioredoxin via the Mechanosensitive Channel MscL during Osmotic Downshock of *Escherichia coli* Cells. J. Biol. Chem..

[13] Kakuda T., Koide Y., Sakamoto A., Takai S. (2012). Characterization of Two Putative Mechanosensitive Channel Proteins of Campylobacter Jejuni Involved in Protection against Osmotic Downshock. Vet. Microbiol..

[14] Chaudhuri R.R., Morgan E., Peters S.E., Pleasance S.J., Hudson D.L., Davies H.M., Wang J., van Diemen P.M., Buckley A.M., Bowen A.J. (2013). Comprehensive Assignment of Roles for *Salmonella* Typhimurium Genes in Intestinal Colonization of Food-Producing Animals. PLoS Genet..

[15] Wang Z., Wang X., Lu P., Ni C., Li Y., van der Veen S. (2018). Identification and Characterization of the *Neisseria gonorrhoeae* MscS-Like Mechanosensitive Channel. Infect. Immun..

[16] Williamson D.R., Dewan K.K., Patel T., Wastella C.M., Ning G., Kirimanjeswara G.S. (2018). A Single Mechanosensitive Channel Protects *Francisella tularensis* Subsp. *Holarctica* from Hypoosmotic Shock and Promotes Survival in the Aquatic Environment. Appl. Environ. Microbiol..

[17] Asogwa M., Miller S., Spano S., Stevens M. (2019). Investigating the Role of the Bacterial Mechanosensitive Channel YnaI in *Salmonella* Pathogenesis. Access Microbiol..

[18] Kouwen T.R.H.M., Trip E.N., Denham E.L., Sibbald M.J.J.B., Dubois J.-Y.F., van Dijl J.M. (2009). The Large Mechanosensitive Channel MscL Determines Bacterial Susceptibility to the Bacteriocin Sublancin 168. Antimicrob. Agents Chemother..

[19] Iscla I., Wray R., Wei S., Posner B., Blount P. (2014). Streptomycin Potency Is Dependent on MscL Channel Expression. Nat. Commun..

[20] Wray R., Iscla I., Gao Y., Li H., Wang J., Blount P. (2016). Dihydrostreptomycin Directly Binds to, Modulates, and Passes through the MscL Channel Pore. PLoS Biol..

[21] Wenzel M., Senges C.H.R., Zhang J., Suleman S., Nguyen M., Kumar P., Chiriac A.I., Stepanek J.J., Raatschen N., May C. (2015). Antimicrobial Peptides from the Aurein Family Form Ion-Selective Pores in *Bacillus subtilis*. ChemBioChem.

[22] Booth I.R., Edwards M.D., Black S., Schumann U., Miller S. (2007). Mechanosensitive Channels in Bacteria: Signs of Closure?. Nat. Rev. Microbiol..

[23] Haswell E.S., Phillips R., Rees D.C. (2011). Mechanosensitive Channels: What Can They Do and How Do They Do It?. Structure.

[24] Li Y. (2002). Ionic Regulation of MscK, a Mechanosensitive Channel from *Escherichia coli*. EMBO J..

[25] Chang G., Spencer R.H., Lee A.T., Barclay M.T., Rees D.C. (1998). Structure of the MscL Homolog from *Mycobacterium tuberculosis*: A Gated Mechanosensitive Ion Channel. Science.

[26] Bass R.B., Strop P., Barclay M., Rees D.C. (2002). Crystal Structure of *Escherichia coli* MscS, a Voltage-Modulated and Mechanosensitive Channel. Science.

[27] Booth I.R. (2014). Bacterial Mechanosensitive Channels: Progress towards an Understanding of Their Roles in Cell Physiology. Curr. Opin. Microbiol..

[28] Nakayama Y., Hashimoto K., Sawada Y., Sokabe M., Kawasaki H., Martinac B. (2018). Corynebacterium Glutamicum Mechanosensitive Channels: Towards Unpuzzling “Glutamate Efflux” for Amino Acid Production. Biophys. Rev..

[29] Kawasaki H., Martinac B. (2020). Mechanosensitive Channels of Corynebacterium Glutamicum Functioning as Exporters of L-Glutamate and Other Valuable Metabolites. Curr. Opin. Chem. Biol..

[30] Cox C.D., Bavi N., Martinac B. (2018). Bacterial Mechanosensors. Annu. Rev. Physiol..

[31] Rasmussen T. (2016). How Do Mechanosensitive Channels Sense Membrane Tension?. Biochem. Soc. Trans..

[32] Iscla I., Blount P. (2012). Sensing and Responding to Membrane Tension: The Bacterial MscL Channel as a Model System. Biophys. J..

[33] Steinbacher S., Bass R., Strop P., Rees D.C., Benos D.J., Simon S.A. (2007). Structures of the Prokaryotic Mechanosensitive Channels MscL and MscS. Current Topics in Membranes.

[34] Reddy B., Bavi N., Lu A., Park Y., Perozo E. (2019). Molecular Basis of Force-from-Lipids Gating in the Mechanosensitive Channel MscS. eLife.

[35] Blount P., Iscla I. (2020). Life with Bacterial Mechanosensitive Channels, from Discovery to Physiology to Pharmacological Target. Microbiol. Mol. Biol. Rev..

[36] Kapsalis C., Wang B., el Mkami H., Pitt S.J., Schnell J.R., Smith T.K., Lippiat J.D., Bode B.E., Pliotas C. (2019). Allosteric Activation of an Ion Channel Triggered by Modification of Mechanosensitive Nano-Pockets. Nat. Commun..

[37] Kapsalis C., Ma Y., Bode B.E., Pliotas C. (2020). In-Lipid Structure of Pressure-Sensitive Domains Hints Mechanosensitive Channel Functional Diversity. Biophys. J..

[38] Naismith J.H., Booth I.R. (2012). Bacterial Mechanosensitive Channels—MscS: Evolution’s Solution to Creating Sensitivity in Function. Annu. Rev. Biophys..

[39] Lu A., Park S., Reddy B., Fei J., Perozo E. (2020). Using Fluorescence Microscopy to Characterize the Role of Mechanosensation in Cell Division. Biophys. J..

[40] Gamini R., Sotomayor M., Chipot C., Schulten K. (2011). Cytoplasmic Domain Filter Function in the Mechanosensitive Channel of Small Conductance. Biophys. J..

[41] Rowe I., Anishkin A., Kamaraju K., Yoshimura K., Sukharev S. (2014). The Cytoplasmic Cage Domain of the Mechanosensitive Channel MscS Is a Sensor of Macromolecular Crowding. J. Gen. Physiol..

[42] Cox C.D., Bavi N., Martinac B. (2019). Biophysical Principles of Ion-Channel-Mediated Mechanosensory Transduction. Cell Rep..

[43] Teng J., Loukin S., Anishkin A., Kung C. (2015). The Force-from-Lipid (FFL) Principle of Mechanosensitivity, at Large and in Elements. Pflügers Arch. Eur. J. Physiol..

[44] Romantsov T., Guan Z., Wood J.M. (2009). Cardiolipin and the Osmotic Stress Responses of Bacteria. Biochim. Biophys. Acta-Biomembr..

[45] Xue F., Cox C.D., Bavi N., Rohde P.R., Nakayama Y., Martinac B. (2020). Membrane Stiffness Is One of the Key Determinants of *E. coli* MscS Channel Mechanosensitivity. Biochim. Biophys. Acta-Biomembr..

[46] Zhang Y., Daday C., Gu R.X., Cox C.D., Martinac B., de Groot B.L., Walz T. (2021). Visualization of the Mechanosensitive Ion Channel MscS under Membrane Tension. Nature.

[47] Anishkin A., Loukin S.H., Teng J., Kung C. (2014). Feeling the Hidden Mechanical Forces in Lipid Bilayer Is an Original Sense. Proc. Natl. Acad. Sci. USA.

[48] Perozo E., Kloda A., Cortes D.M., Martinac B. (2002). Physical Principles Underlying the Transduction of Bilayer Deformation Forces during Mechanosensitive Channel Gating. Nat. Struct. Biol..

[49] Nomura T., Cranfield C.G., Deplazes E., Owen D.M., Macmillan A., Battle A.R., Constantine M., Sokabe M., Martinac B. (2012). Differential Effects of Lipids and Lyso-Lipids on the Mechanosensitivity of the Mechanosensitive Channels MscL and MscS. Proc. Natl. Acad. Sci. USA.

[50] Carniello V., Peterson B.W., van der Mei H.C., Busscher H.J. (2020). Role of Adhesion Forces in Mechanosensitive Channel Gating in *Staphylococcus aureus* Adhering to Surfaces. NPJ Biofilms Microbiomes.

[51] Li J., Busscher H.J., Swartjes J.J.T.M., Chen Y., Harapanahalli A.K., Norde W., van der Mei H.C., Sjollema J. (2014). Residence-Time Dependent Cell Wall Deformation of Different *Staphylococcus aureus* Strains on Gold Measured Using Surface-Enhanced-Fluorescence. Soft Matter.

[52] Gu J., Valdevit A., Chou T.M., Libera M. (2017). Substrate Effects on Cell-Envelope Deformation during Early-Stage: *Staphylococcus aureus* Biofilm Formation. Soft Matter.

[53] Culham D.E., Dalgado C., Gyles C.L., Mamelak D., MacLellan S., Wood J.M. (1998). Osmoregulatory Transporter ProP Influences Colonization of the Urinary Tract by *Escherichia coli*. Microbiology.

[54] Maurin M., Gyuranecz M. (2016). Tularaemia: Clinical Aspects in Europe. Lancet Infect. Dis..

[55] Sjöstedt A. (2007). Tularemia: History, Epidemiology, Pathogen Physiology, and Clinical Manifestations. Ann. N. Y. Acad. Sci..

[56] Jackson J., McGregor A., Cooley L., Ng J., Brown M., Ong C.W., Darcy C., Sintchenko V. (2012). *Francisella tularensis* Subspecies *Holarctica*, Tasmania, Australia, 2011. Emerg. Infect. Dis..

[57] Igwaran A., Okoh A.I. (2019). Human Campylobacteriosis: A Public Health Concern of Global Importance. Heliyon.

[58] Lawe-Davies O., Bennett S., WHO (2017). WHO Publishes List of Bacteria for Which New Antibiotics Are Urgently Needed. Saudi Med. J..

[59] Ipe D.S., Horton E., Ulett G.C. (2016). The Basics of Bacteriuria: Strategies of Microbes for Persistence in Urine. Front. Cell. Infect. Microbiol..

[60] Schwan W.R. (2009). Survival of Uropathogenic *Escherichia coli* in the Murine Urinary Tract Is Dependent on OmpR. Microbiology.

[61] Gargan R.A., Hamilton-Miller J.M., Brumfitt W. (1993). Effect of PH and Osmolality on in Vitro Phagocytosis and Killing by Neutrophils in Urine. Infect. Immun..

[62] Mittal R., Sharma S., Chhibber S., Harjai K. (2009). Effect of Osmolarity on Virulence of Uropathogenic *Pseudomonas aeruginosa*. Am. J. Biomed. Sci..

[63] Wray R., Iscla I., Blount P. (2021). Curcumin Activation of a Bacterial Mechanosensitive Channel Underlies Its Membrane Permeability and Adjuvant Properties. PLoS Pathog..

[64] Wray R., Herrera N., Iscla I., Wang J., Blount P. (2019). An Agonist of the MscL Channel Affects Multiple Bacterial Species and Increases Membrane Permeability and Potency of Common Antibiotics. Mol. Microbiol..

[65] Wray R., Wang J., Iscla I., Blount P. (2020). Novel MscL Agonists That Allow Multiple Antibiotics Cytoplasmic Access Activate the Channel through a Common Binding Site. PLoS ONE.

[66] Oswald F., Varadarajan A., Lill H., Peterman E.J.G., Bollen Y.J.M. (2016). MreB-Dependent Organization of the *E. coli* Cytoplasmic Membrane Controls Membrane Protein Diffusion. Biophys. J..

[67] Becker M., Börngen K., Nomura T., Battle A.R., Marin K., Martinac B., Krämer R. (2013). Glutamate Efflux Mediated by Corynebacterium Glutamicum MscCG, *Escherichia coli* MscS, and Their Derivatives. Biochim. Biophys. Acta-Biomembr..

[68] Wang Y., Cao G., Xu D., Fan L., Wu X., Ni X., Zhao S., Zheng P., Sun J., Ma Y. (2018). A Novel Corynebacterium Glutamicum l-Glutamate Exporter. Appl. Environ. Microbiol..

[69] Kaldalu N., Mei R., Lewis K. (2004). Killing by Ampicillin and Ofloxacin Induces Overlapping Changes in *Escherichia coli* Transcription Profile. Antimicrob. Agents Chemother..

[70] Mathieu A., Fleurier S., Frénoy A., Dairou J., Bredeche M.-F., Sanchez-Vizuete P., Song X., Matic I. (2016). Discovery and Function of a General Core Hormetic Stress Response in *E. coli* Induced by Sublethal Concentrations of Antibiotics. Cell Rep..

[71] Jiafeng L., Fu X., Chang Z. (2015). Hypoionic Shock Treatment Enables Aminoglycosides Antibiotics to Eradicate Bacterial Persisters. Sci. Rep..

[72] Zhao Y., Lv B., Sun F., Liu J., Wang Y., Gao Y., Qi F., Chang Z., Fu X. (2020). Rapid Freezing Enables Aminoglycosides to Eradicate Bacterial Persisters via Enhancing Mechanosensitive Channel MscL-Mediated Antibiotic Uptake. mBio.

[73] Nakamura J., Hirano S., Ito H., Wachi M. (2007). Mutations of the Corynebacterium Glutamicum NCgl1221 Gene, Encoding a Mechanosensitive Channel Homolog, Induce l-Glutamic Acid Production. Appl. Environ. Microbiol..

[74] Hashimoto K., Murata J., Konishi T., Yabe I., Nakamatsu T., Kawasaki H. (2012). Glutamate Is Excreted Across the Cytoplasmic Membrane through the NCgl1221 Channel of Corynebacterium Glutamicum by Passive Diffusion. Biosci. Biotechnol. Biochem..

[75] Hirasawa T., Saito M., Yoshikawa K., Furusawa C., Shmizu H. (2018). Integrated Analysis of the Transcriptome and Metabolome of Corynebacterium Glutamicum during Penicillin-Induced Glutamic Acid Production. Biotechnol. J..

[76] Koprowski P., Grajkowski W., Balcerzak M., Filipiuk I., Fabczak H., Kubalski A. (2015). Cytoplasmic Domain of MscS Interacts with Cell Division Protein FtsZ: A Possible Non-Channel Function of the Mechanosensitive Channel in *Escherichia coli*. PLoS ONE.

[77] Yang J.H., Wright S.N., Hamblin M., McCloskey D., Alcantar M.A., Schrübbers L., Lopatkin A.J., Satish S., Nili A., Palsson B.O. (2019). A White-Box Machine Learning Approach for Revealing Antibiotic Mechanisms of Action. Cell.

[78] Matsuzaki K., Harada M., Funakoshi S., Fujii N., Miyajima K. (1991). Physicochemical Determinants for the Interactions of Magainins 1 and 2 with Acidic Lipid Bilayers. Biochim. Biophys. Acta-Biomembr..

[79] Dürr U.H.N., Sudheendra U.S., Ramamoorthy A. (2006). LL-37, the Only Human Member of the Cathelicidin Family of Antimicrobial Peptides. Biochim. Biophys. Acta-Biomembr..

[80] Kandasamy S.K., Larson R.G. (2006). Effect of Salt on the Interactions of Antimicrobial Peptides with Zwitterionic Lipid Bilayers. Biochim. Biophys. Acta-Biomembr..

[81] Wu G., Ding J., Li H., Li L., Zhao R., Shen Z., Fan X., Xi T. (2008). Effects of Cations and PH on Antimicrobial Activity of Thanatin and S-Thanatin against *Escherichia coli* ATCC25922 and *B. subtilis* ATCC 21332. Curr. Microbiol..

[82] Aoki W., Ueda M. (2013). Characterization of Antimicrobial Peptides toward the Development of Novel Antibiotics. Pharmaceuticals.

[83] Wahid M.H., Stroeher U.H., Eroglu E., Chen X., Vimalanathan K., Raston C.L., Boulos R.A. (2015). Aqueous Based Synthesis of Antimicrobial-Decorated Graphene. J. Colloid Interface Sci..

[84] Wright L., Rao S., Thomas N., Boulos R.A., Prestidge C.A. (2018). Ramizol ^®^ Encapsulation into Extended Release PLGA Micro- and Nanoparticle Systems for Subcutaneous and Intramuscular Administration: In Vitro and in Vivo Evaluation. Drug Dev. Ind. Pharm..

[85] Sibley K., Chen J., Koetzner L., Mendes O., Kimzey A., Lansita J., Boulos R.A. (2019). A 14-Day Repeat Dose Oral Gavage Range-Finding Study of a First-in-Class CDI Investigational Antibiotic, in Rats. Sci. Rep..

[86] Wolfe C., Pagano P., Pillar C.M., Shinabarger D.L., Boulos R.A. (2018). Comparison of the in Vitro Antibacterial Activity of Ramizol, Fidaxomicin, Vancomycin, and Metronidazole against 100 Clinical Isolates of Clostridium Difficile by Broth Microdilution. Diagn. Microbiol. Infect. Dis..

[87] Rao S., Prestidge C.A., Miesel L., Sweeney D., Shinabarger D.L., Boulos R.A. (2016). Preclinical Development of Ramizol, an Antibiotic Belonging to a New Class, for the Treatment of *Clostridium difficile* Colitis. J. Antibiot..

[88] Iscla I., Wray R., Blount P., Larkins-Ford J., Conery A.L., Ausubel F.M., Ramu S., Kavanagh A., Huang J.X., Blaskovich M.A. (2015). A New Antibiotic with Potent Activity Targets MscL. J. Antibiot..

[89] Wray R., Iscla I., Kovacs Z., Wang J., Blount P. (2019). Novel Compounds That Specifically Bind and Modulate MscL: Insights into Channel Gating Mechanisms. FASEB J..

[90] Wray R., Blount P., Wang J., Iscla I. (2022). In Silico Screen Identifies a New Family of Agonists for the Bacterial Mechanosensitive Channel MscL. Antibiotics.

[91] Nguyen T., Clare B., Guo W., Martinac B. (2005). The Effects of Parabens on the Mechanosensitive Channels of *E. coli*. Eur. Biophys. J..

[92] Kamaraju K., Sukharev S. (2008). The Membrane Lateral Pressure-Perturbing Capacity of Parabens and Their Effects on the Mechanosensitive Channel Directly Correlate with Hydrophobicity. Biochemistry.

[93] Comert F., Greenwood A., Maramba J., Acevedo R., Lucas L., Kulasinghe T., Cairns L.S., Wen Y., Fu R., Hammer J. (2019). The Host-Defense Peptide Piscidin P1 Reorganizes Lipid Domains in Membranes and Decreases Activation Energies in Mechanosensitive Ion Channels. J. Biol. Chem..

[94] Cetuk H., Maramba J., Britt M., Scott A.J., Ernst R.K., Mihailescu M., Cotten M.L., Sukharev S. (2020). Differential Interactions of Piscidins with Phospholipids and Lipopolysaccharides at Membrane Interfaces. Langmuir.

[95] Hurst A.C., Gottlieb P.A., Martinac B. (2009). Concentration Dependent Effect of GsMTx4 on Mechanosensitive Channels of Small Conductance in *E. coli* Spheroplasts. Eur. Biophys. J..

[96] Kamaraju K., Gottlieb P.A., Sachs F., Sukharev S. (2010). Effects of GsMTx4 on Bacterial Mechanosensitive Channels in Inside-Out Patches from Giant Spheroplasts. Biophys. J..

[97] Jung H.J., Kim P.I., Lee S.K., Lee C.W., Eu Y.J., Lee D.G., Earm Y.E., Kim J.I. (2006). Lipid Membrane Interaction and Antimicrobial Activity of GsMTx-4, an Inhibitor of Mechanosensitive Channel. Biochem. Biophys. Res. Commun..

[98] Berrier C., Coulombe A., Szabo I., Zoratti M., Ghazi A. (1992). Gadolinium Ion Inhibits Loss of Metabolites Induced by Osmotic Shock and Large Stretch-activated Channels in Bacteria. Eur. J. Biochem..

[99] Ermakov Y.A., Kamaraju K., Sengupta K., Sukharev S. (2010). Gadolinium Ions Block Mechanosensitive Channels by Altering the Packing and Lateral Pressure of Anionic Lipids. Biophys. J..

[100] Martinac B., Adler J., Kung C. (1990). Mechanosensitive Ion Channels of *E. coli* Activated by Amphipaths. Nature.

[101] Atcha H., Jairaman A., Holt J.R., Meli V.S., Nagalla R.R., Veerasubramanian P.K., Brumm K.T., Lim H.E., Othy S., Cahalan M.D. (2021). Mechanically Activated Ion Channel Piezo1 Modulates Macrophage Polarization and Stiffness Sensing. Nat. Commun..

[102] Beaulieu-Laroche L., Christin M., Donoghue A., Agosti F., Yousefpour N., Petitjean H., Davidova A., Stanton C., Khan U., Dietz C. (2020). TACAN Is an Ion Channel Involved in Sensing Mechanical Pain. Cell.

[103] Tran D., Petitjean H., Chebli Y., Geitmann A., Sharif-Naeini R. (2021). Mechanosensitive Ion Channels Contribute to Mechanically Evoked Rapid Leaflet Movement in *Mimosa pudica*. Plant Physiol..

[104] Parisi C., Chandaria V.V., Nowlan N.C. (2018). Blocking Mechanosensitive Ion Channels Eliminates the Effects of Applied Mechanical Loading on Chick Joint Morphogenesis. Philos. Trans. R. Soc. B.

[105] Gu Y., Gu C. (2014). Physiological and Pathological Functions of Mechanosensitive Ion Channels. Mol. Neurobiol..

[106] Martinac B., Nomura T., Chi G., Petrov E., Rohde P.R., Battle A.R., Foo A., Constantine M., Rothnagel R., Carne S. (2014). Bacterial Mechanosensitive Channels: Models for Studying Mechanosensory Transduction. Antioxid. Redox Signal..

[107] Herrera N., Maksaev G., Haswell E.S., Rees D.C. (2018). Elucidating a Role for the Cytoplasmic Domain in the Mycobacterium Tuberculosis Mechanosensitive Channel of Large Conductance. Sci. Rep..

[108] Wang W., Black S.S., Edwards M.D., Miller S., Morrison E.L., Bartlett W., Dong C., Naismith J.H., Booth I.R. (2008). The Structure of an Open Form of an *E. coli* Mechanosensitive Channel at 3.45 å Resolution. Science.

[109] Rasmussen T., Flegler V.J., Rasmussen A., Böttcher B. (2019). Structure of the Mechanosensitive Channel MscS Embedded in the Membrane Bilayer. J. Mol. Biol..

[110] Pliotas C., Ward R., Branigan E., Rasmussen A., Hagelueken G., Huang H., Black S.S., Booth I.R., Schiemann O., Naismith J.H. (2012). Conformational State of the MscS Mechanosensitive Channel in Solution Revealed by Pulsed Electron-Electron Double Resonance (PELDOR) Spectroscopy. Proc. Natl. Acad. Sci. USA.

[111] Dorwart M.R., Wray R., Brautigam C.A., Jiang Y., Blount P.S. (2010). Aureus MscL Is a Pentamer In Vivo but of Variable Stoichiometries In Vitro: Implications for Detergent-Solubilized Membrane Proteins. PLoS Biol..

[112] Liu Z., Gandhi C.S., Rees D.C. (2009). Structure of a Tetrameric MscL in an Expanded Intermediate State. Nature.

[113] Pliotas C., Dahl A.C.E., Rasmussen T., Mahendran K.R., Smith T.K., Marius P., Gault J., Banda T., Rasmussen A., Miller S. (2015). The Role of Lipids in Mechanosensation. Nat. Struct. Mol. Biol..

[114] Angiulli G., Dhupar H.S., Suzuki H., Wason I.S., Duong Van Hoa F., Walz T. (2020). New Approach for Membrane Protein Reconstitution into Peptidiscs and Basis for Their Adaptability to Different Proteins. eLife.

[115] Flegler V.J., Rasmussen A., Borbil K., Boten L., Chen H.A., Deinlein H., Halang J., Hellmanzik K., Löffler J., Schmidt V. (2021). Mechanosensitive Channel Gating by Delipidation. Proc. Natl. Acad. Sci. USA.

[116] Yu J., Zhang B., Zhang Y., Xu C.-Q., Zhuo W., Ge J., Li J., Gao N., Li Y., Yang M. (2018). A Binding-Block Ion Selective Mechanism Revealed by a Na/K Selective Channel. Protein Cell.

[117] Zhang X., Wang J., Feng Y., Ge J., Li W., Sun W., Iscla I., Yu J., Blount P., Li Y. (2012). Structure and Molecular Mechanism of an Anion-Selective Mechanosensitive Channel of Small Conductance. Proc. Natl. Acad. Sci. USA.

[118] Lai J.Y., Poon Y.S., Kaiser J.T., Rees D.C. (2013). Open and Shut: Crystal Structures of the Dodecylmaltoside Solubilized Mechanosensitive Channel of Small Conductance from *Escherichia coli* and *Helicobacter pylori* at 4.4 Å and 4.1 Å Resolutions. Protein Sci..

[119] Flegler V.J., Rasmussen A., Rao S., Wu N., Zenobi R., Sansom M.S.P., Hedrich R., Rasmussen T., Böttcher B. (2020). The MscS-like Channel YnaI Has a Gating Mechanism Based on Flexible Pore Helices. Proc. Natl. Acad. Sci. USA.

[120] Catalano C., Ben-Hail D., Qiu W., Blount P., des Georges A., Guo Y. (2021). Cryo-EM Structure of Mechanosensitive Channel YnaI Using SMA2000: Challenges and Opportunities. Membranes.

[121] Hu W., Wang Z., Zheng H. Extended Sensor Paddles with Bound Lipids Revealed in Mechanosensitive Channel YnaI.

[122] Walton T.A., Rees D.C. (2013). Structure and Stability of the C-Terminal Helical Bundle of the *E. coli* Mechanosensitive Channel of Large Conductance. Protein Sci..

[123] Wu J., Ke M. Mechanosensitive Channel MscS K180R Mutant. https://www.rcsb.org/structure/7DLU.

