# Supplementary material for: Roles of Bacterial Mechanosensitive Channels in Infection and Antibiotic Susceptibility
Source: Pharmaceuticals (Basel). 2022 Jun 21;15(7):770. doi: 10.3390/ph15070770 (PMC9322971; doi:10.3390/ph15070770)
Supplement: Supplementary file 1 [file pharmaceuticals-15-00770-s001.zip › pharmaceuticals-1745286-supplementary.pdf]

**Supplementary Table S1:** Available PDB structures of mechanosensitive channels.

| **Protein name** | **PDB number** | **Organism** | **Structural family** | **Remarks** | **Reference** |
|---|---|---|---|---|---|
| Mechanosensitive channel of small conductance (MscS) | 2OAU | *E. coli* | MscS | Version 1.2 | [33] |
| Mechanosensitive channel of large conductance (MscL) | 2OAR | *M. tuberculosis* | MscL | Version 1.3 | |
| C-terminal domain truncation of the *Mycobacterium tuberculosis* mechanosensitive channel of large conductance MscL | 6CTD | *M. tuberculosis* | MscL | Version 1.1 | [107] |
| Open structure of MscS | 2VV5 | *E. coli* | MscS | Version 1.1 | [108] |
| Cryo-EM structure of mechanosensitive channel MscS in PC-10 nanodiscs | 6VYL | *E. coli* | MscS | Version1.2<br>Related structure<br>6VYK, 6VYL, 6VYM | [46] |
| Cryo-EM structure of mechanosensitive channel MscS in PC-18:1 nanodiscs treated with beta-cyclodextran | 6VYM | *E. coli* | MscS | Version 1.2 | |
| Cryo-EM structure of mechanosensitive channel MscS in PC-18:1 nanodiscs | 6VYK | *E. coli* | MscS | Version 1.2 | |
| MscS Nanodisc | 6PWP | *E. coli* | MscS | Version 1.0<br>Related structure 6PWN, 6PWO, 6PWP | [34] |
| MscS Nanodisc with N-terminal His-tag | 6PWN | *E. coli* | MscS | Version 1.0 | |
| Mscs DDM | 6PWO | *E. coli* | MscS | Version 1.0 | |
| Structure of the mechanosensitive channel MscS embedded in the membrane bilayer | 6RLD | *E. coli* | MscS | Version 1.2 | [109] |
| MTSSL spin labeled D67C mutant of MscS in the open form | 4AGE | *E. coli* | MscS | Version 1.2 | [110] |

| | | | | | |
|---|---|---|---|---|---|
| | | | | Related Structures:<br>4AGE, 4AGF | |
| MTSSL spin labeled L124C mutant of MscS in the open form | 4GF | *E. coli* | MscS | Version 1.2 | |
| Structure of a tetrameric MscL in an expanded intermediate state | 3HZQ | *S. aureus* | MscL | Version 1.3<br>see also: *S. aureus* MscL is pentameric *in vivo*[111] | [112] |
| MscS D67R1 high resolution | 5AJI | *E. coli* | MscS | Version 1.2 | [113] |
| Cryo-EM structure of mechanosensitive channel MscS reconstituted into peptidiscs | 6UZH | *E. coli* | MscS | Version 1.1 | [114] |
| Mechanosensitive channel MscS solubilized with DDM in closed conformation | 7ONL | *E. coli* | MscS | Version 1.0<br>Related structure:<br>7ONJ, 7ONL, 7OO0, 7OO6, 7OO8, 7OOA | [115] |
| Mechanosensitive channel MscS solubilized with DDM in closed conformation with added lipid | 7OO6 | *E. coli* | MscS | Version 1.0 | |
| Mechanosensitive channel MscS solubilized with DDM in open conformation | 7OO0 | *E. coli* | MscS | Version 1.0 | |
| Mechanosensitive channel MscS solubilized with LMNG in open conformation | 7ONJ | *E. coli* | MscS | Version 1.0 | |
| Mechanosensitive channel MscS solubilized with LMNG in open conformation with added lipid | 7OOA | *E. coli* | MscS | Version 1.0 | |
| Mechanosensitive channel MscS solubilized with LMNG in closed conformation with added lipid | 7OO8 | *E. coli* | MscS | Version 1.0 | |
| Cryo-EM structure of MscS channel YnaI | 5Y4O | *E. coli* | MscS | Version 1.0 | [116] |
| Crystal structure of a membrane protein | 3UDC | *Caldanaerobacter subterraneus* | MscS | Version 1.2<br>Related structure:<br>3T9N, 3UDC | [117] |

| | | | | | |
|---|---|---|---|---|---|
| Crystal structure of a membrane protein | 3T9N | *Caldanaerobacter subterraneus* | MscS | Version 1.1 | |
| Crystal structure of *Helicobacter pylori* MscS (closed state) | 4HW9 | *H. pylori* | MscS | Version 1.2<br>Related Structures:<br>4HW9, 4HWA | [118] |
| Crystal Structure of *E. coli* MscS wild type (open state) | 4HWA | *E. coli* | MscS | Version 1.1 | |
| small conductance mechanosensitive channel YbiO | 7A46 | *E. coli* | MscS | Version 1.1<br>Related Structures:<br>6ZYD, 6ZYE, 7A46 | [119] |
| YnaI | 6ZYD | *E. coli* | MscS | Version 1.0 | |
| YnaI in an open-like conformation | 6ZYE | *E. coli* | MscS | Version 1.0 | |
| Low conductance mechanosensitive channel YnaI | 7N4T | *E. coli* | MscS | Version 1.0 | [120] |
| Extended sensor paddles with bound lipids revealed in mechanosensitive channel YnaI | 6URT | *E. coli* | MscS | Version 1.0 | [121] |
| Structure of the C-terminal domain of the *E. coli* mechanosensitive channel of large conductance | 4LKU | *E. coli* | MscL | Version 1.2 | [122] |
| Mechanosensitive channel MscS K180R mutant | 7DLU | *P. aeruginosa* | MscS | Version 1.0 | [123] |
